# Interspecific interactions within a vector-borne complex are influenced by a co-occurring pathosystem

**DOI:** 10.1038/s41598-021-81710-w

**Published:** 2021-01-26

**Authors:** Regina K. Cruzado-Gutiérrez, Rohollah Sadeghi, Sean M. Prager, Clare L. Casteel, Jessica Parker, Erik J. Wenninger, William J. Price, Nilsa A. Bosque-Pérez, Alexander V. Karasev, Arash Rashed

**Affiliations:** 1 Department of Entomology, Plant Pathology and Nematology, University of Idaho, Aberdeen R&E Center, Aberdeen, ID 83210 USA; 2 Department of Entomology, Plant Pathology and Nematology, University of Idaho, Moscow, ID 83844 USA; 3 Department of Plant Science, University of Saskatchewan, Saskatoon, SK S7N 5A8 Canada; 4 Department of Plant Pathology and Plant-Microbe Biology, Cornell University, Ithaca, NY 14853 USA; 5 Department of Entomology, Plant Pathology and Nematology, Kimberly Research & Extension Center, University of Idaho, Kimberly, ID 83341 USA; 6 College of Agricultural and Life Sciences, Statistical Programs, University of Idaho, Moscow, ID 83844 USA

**Keywords:** Ecological epidemiology, Ecophysiology, Ecology

## Abstract

*Potato virus Y* (PVY) and zebra chip (ZC) disease are major threats to solanaceous crop production in North America. PVY can be spread by aphid vectors and through vegetative propagation in potatoes. ZC is associated with “*Candidatus* Liberibacter solanacearum” (Lso), which is transmitted by the tomato/potato psyllid, *Bactericera cockerelli* Šulc (Hemiptera: Triozidae). As these two pathosystems may co-occur, we studied whether the presence of one virus strain, PVY°, affected the host preference, oviposition, and egg hatch rate of Lso-free or Lso-carrying psyllids in tomato plants. We also examined whether PVY infection influenced Lso transmission success by psyllids, Lso titer and plant chemistry (amino acids, sugars, and phytohormones). Lso-carrying psyllids showed a preference toward healthy hosts, whereas the Lso-free psyllids preferentially settled on the PVY-infected tomatoes. Oviposition of the Lso-carrying psyllids was lower on PVY-infected than healthy tomatoes, but Lso transmission, titer, and psyllid egg hatch were not significantly affected by PVY. The induction of salicylic acid and its related responses, and not nutritional losses, may explain the reduced attractiveness of the PVY-infected host to the Lso-carrying psyllids. Although our study demonstrated that pre-existing PVY infection can reduce oviposition by the Lso-carrying vector, the preference of the Lso-carrying psyllids to settle on healthy hosts could contribute to Lso spread to healthy plants in the presence of PVY infection in a field.

## Introduction

Pathogens are known to manipulate their host plants in ways that alter vector preference, performance, and subsequent pathogen spread^1,2^. Alterations in visual and olfactory cues^3–7^ and/or host plant defense responses^8–10^ are some of the pathogen-induced plant changes that can affect insect behavior and life history traits^10–12^. Although plant-vector-pathogen interactions have been the subject of many studies, research on interactions between or among vector-borne pathosystems remain limited, even though under natural circumstances pest conditions can be sympatric, and in many instances, they share host plants.

Interactions between co-infecting pathogens can be synergistic, antagonistic or neutral^13–15^. A classic example of synergism is reported between *Potato virus Y* (PVY, genus *Potyvirus*) and *Potato virus X* (PVX, genus *Potexvirus*), where the co-infection resulted in increased disease severity compared to single viral infections. Vance^13^ showed that following coinfection, PVX titer increased whereas PVY titer remained constant. This increase in the PVX titer is in part due to the suppression of RNA gene silencing in the host plant by a viral protein encoded by PVY^16^. Antagonistic interactions occur when one pathogen negatively impacts development of the other(s)^17,18^. For example, it has been demonstrated that in co-infected rice, an increase in the bacterium *Xanthomonas oryzae* resulted in a decrease in *Rice yellow mottle virus* titer^18^. In tomato plants, co-infection of tomato big bud phytoplasma and *Tomato yellow leaf curl virus* hinders multiplication of either pathogen, which could potentially reduce infection risks and losses in greenhouses^19^.

Pre-existing pathogen infections can also affect dynamics of vector-borne plant pathogens through changes in vector behavior, oviposition and transmission. *Tobacco mosaic virus* (TMV) infection was shown to reduce tomato attractiveness to the tomato/potato psyllid (*Bactericera cockerelli* [Hem., Triozidae]) vector of “*Candidatus* Liberibacter solanacearum” (Lso)^20^. Shapiro et al.^21^ provided another example in which plants infected with *Zucchini yellow mosaic virus* became less attractive to *Acalymma vittatum*, the beetle vector of the bacterial pathogen *Erwinia tracheiphlia*, resulting in a reduced incidence of the *Erwinia* in *Zucchini yellow mosaic virus*-infected plants.

Vector-borne pathosystems represent key arenas for “crosstalk” among signaling and defense. Salicylic acid (SA), jasmonic acid (JA), ethylene (ET), and abscisic acid (ABA) are known to be essential mediators for plant immune responses. While SA is primarily known as a host resistance regulator against pathogens^22^, JA is a major signaling compound in plant defense against herbivores^22,23^. Further, phloem-feeding insects such as whiteflies and aphids may induce both SA-dependent and JA/ET-dependent signaling pathways^24^. It is well established that SA signaling can antagonize JA^22,24–26^ signaling, whereas ABA and ET can have either synergistic or antagonist interaction with JA^22,27,28^ and SA^23,29–31^. Pathogen induction of SA can potentially benefit vectors and other herbivores through inhibition of JA defense responses, as has been demonstrated in *Tomato yellow leaf curl virus* (vector: *Bemisia tabaci*)^6^ and *Tomato spotted wilt virus* (vector: *Frankliniella occidentalis*)^10,32^. Although several molecular mechanisms underlying plant–herbivore interactions have been determined^33^, the induction of phytohormone-related resistance is context-dependent due to spatial and temporal interactions among phytohormone^23^.

Plant primary metabolites such as amino acids and sugars are known to shift in response to pathogenic infections. Several studies have demonstrated reduced sugar concentrations in response to pathogens^20,34^. For example, Prager et al.^20^ showed that the tomato plants infected with TMV contain lower concentrations of reducing sugars (i.e., fructose and glucose) compared to their healthy counterparts. There are also studies in which increases in some but not all sugars following viral infections have been documented^35,36^. Plant viruses can also induce changes in the amino acid contents of phloem^34^ and/or plant leaves^37,38^. Changes in free amino acids^34,37,38^, sugar concentrations^35,39^, or soluble carbohydrate content in leaves^40^ could influence the behavior of the vector, subsequently impacting pathogen spread^1,41^. While pathogen-infected host plants with elevated defenses may not be optimal resources for herbivores, they may be preferred hosts if higher concentrations of key nutrients are available in infected tissue^42^.

Lso and PVY are two pathogens that frequently infect a wide range of solanaceous crops such as tomato (*Solanum lycopersicum*), pepper (*Capsicum annuum*), eggplant (*Solanum melongena*), and potato (*Solanum tuberosum*)^43,44^. Lso and PVY infections can each cause yellowing, necrosis, and foliar deformations as well as yield and quality losses in tomato and potato, rendering fruits and tubers unmarketable^44–47^. It is also important to note that symptoms of PVY infection in solanaceous hosts may widely vary with the strain of the virus^44,48^. Most of the characterized PVY isolates can be classified as either potato or pepper-adapted, with tomato being an intermediate permissive hosts for both types^44,45,48,49^. Although PVY is known to be transmitted by many species of aphids in a non-persistent manner, Lso is transmitted by the tomato/potato psyllid persistently^50^. In the Western United States, Lso-carrying psyllids usually arrive later during the growing season when PVY infections are already established^51,52^. The goal of this study was to determine the effects of PVY (strain PVY°) infection on interactions among Lso, the tomato/potato psyllid vector of Lso, and a host shared by both pathogens. To address this, we examined Lso inoculation success and titer in the presence and absence of PVY infection. Also, the host preference, oviposition, and hatch rate of Lso-carrying and Lso-free tomato/potato psyllids were analyzed in the presence of pre-existing PVY infection in the tomato host. Moreover, to explain any potential effect of PVY-induced changes in plant chemistry, we measured amino acids and sugars, as well as SA, JA, and ABA concentrations in PVY-infected and healthy hosts.

## Results

### Effect of PVY infection on inoculation success of “*Ca.* Liberibacter solanacearum” by psyllids

**Figure 1:**
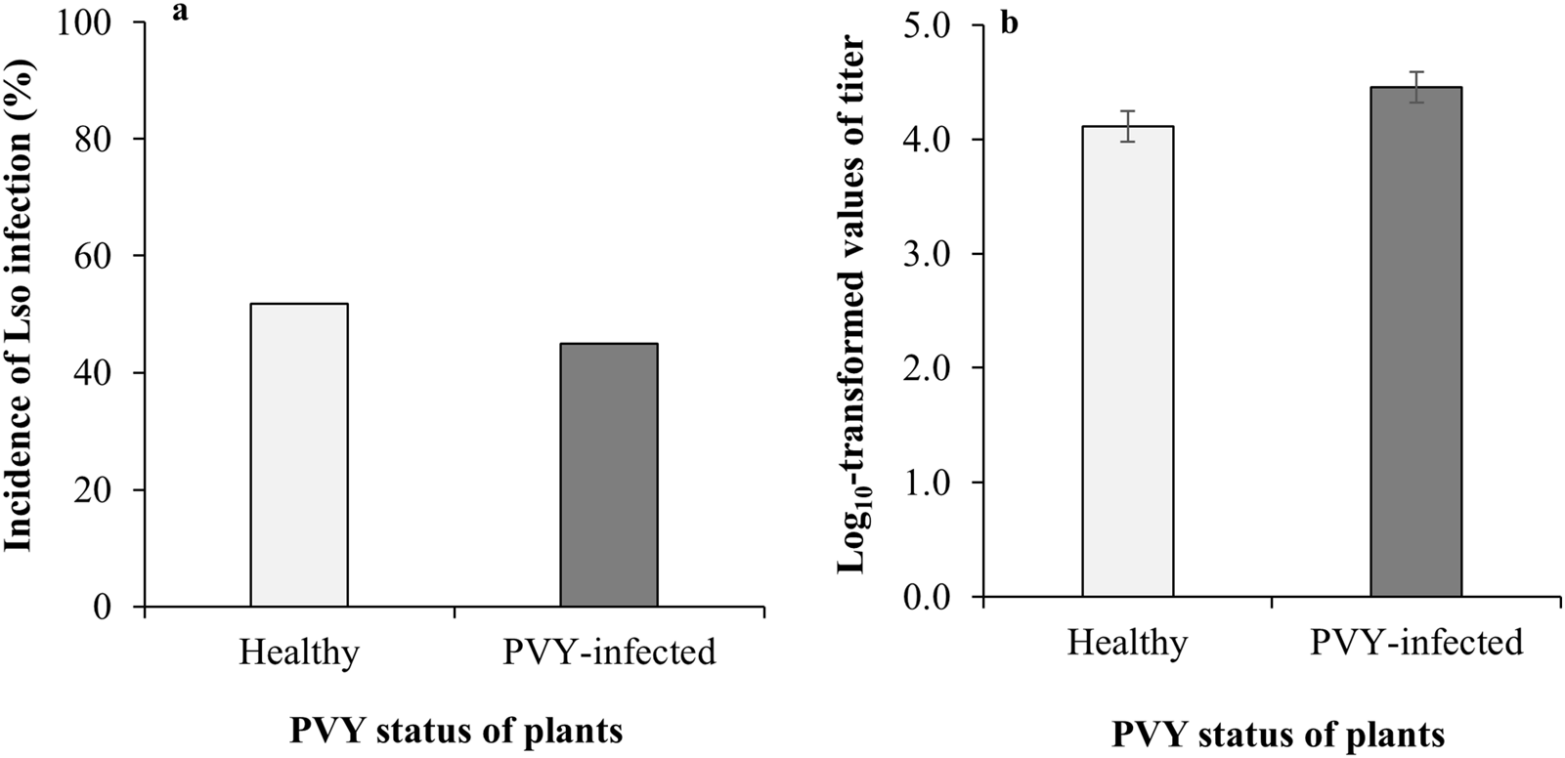
“*Candidatus* Liberibacter solanacerum” in healthy and PVY-infected tomato plants demonstrating no significant difference in either (**a**) transmission success across time-blocks, or (**b**) titers. Error bars represent standard error (± SE).

Lso inoculation success by the tomato/potato psyllids was not significantly affected by the PVY presence in the tomato host plant (GLMM, F_1,67_ = 5.12, *P* = 0.477, Fig. 1a). Similar to inoculation success, Lso titer in the tomato plant tissue was not significantly affected by the PVY infection status of the host (GLMM, F_1,31_ = 1.08, *P* = 0.305) (Fig. 1b).

### The effect of PVY infection on psyllid oviposition and egg hatch rate

**Figure 2:**
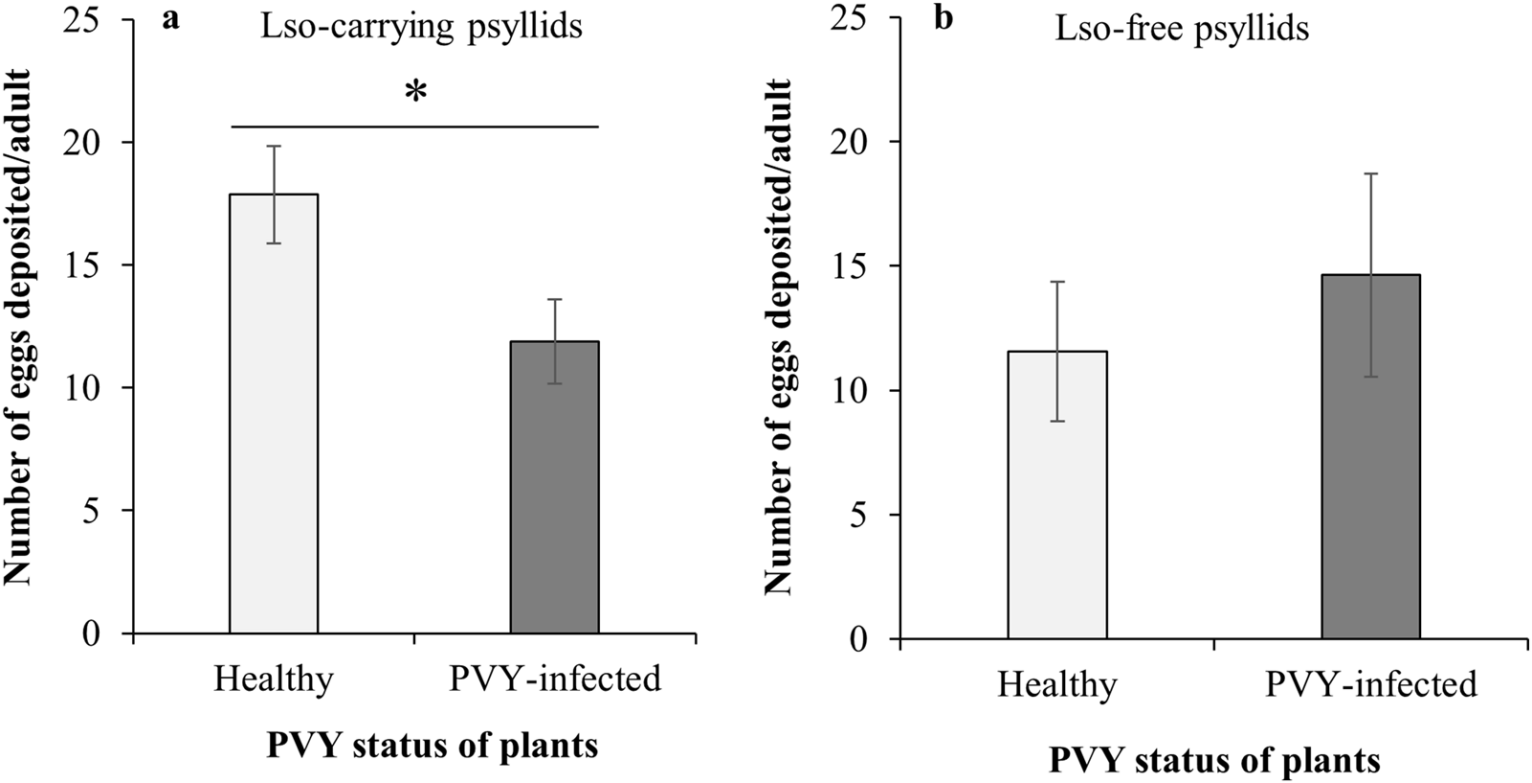
Oviposition of tomato/potato psyllids on healthy and PVY-infected tomato plants. (**a**) Lso-infected tomato/potato psyllids (**b**) Lso-free tomato/potato psyllids. Significant difference in number of eggs laid is indicated by asterisk (*P* ≤ 0.05). Error bars represent standard error (± SE).

Oviposition of the Lso-carrying psyllids was significantly lower on the PVY-infected compared to the healthy tomatoes (Mann–Whitney, *U* = 415.0, *n*_1_ = 43, *n*_2_ = 30, *P* = 0.010) (Fig. 2a). However, no significant effect of PVY presence was detected for the oviposition of the Lso-free tomato/potato psyllids (Mann–Whitney, *U* = 242.0, *n*_1_ = 24, *n*_2_ = 19, *P* = 0.731) (Fig. 2b). The hatch rate of Lso-carrying psyllid eggs was not significantly different between healthy (69.9%) and PVY-infected (70.5%) plants (GLMM, F_1,63_ = 0.007, *P* = 0.931). Likewise, the hatch rate of Lso-free psyllid eggs was not significantly affected by the presence of PVY (GLMM, F_1,34_ = 0.170, *P* = 0.683); Lso-free psyllid eggs showed a hatch rate of 60.7% and 64.6% on healthy and PVY-infected plants, respectively.

### The effect of PVY infection on psyllid host preference

**Figure 3:**
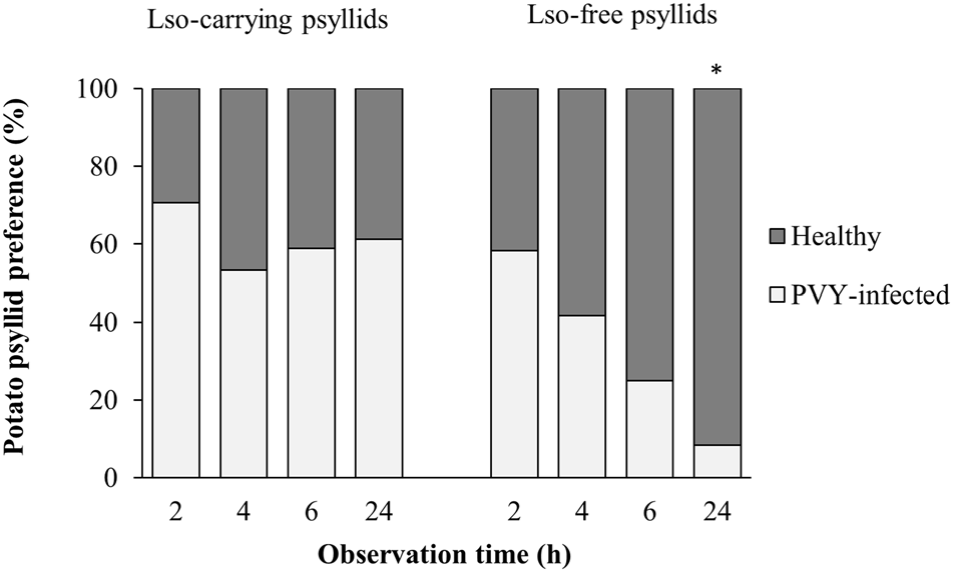
Percentage of Lso-carrying and Lso-free psyllids observed on healthy and PVY-infected tomato plants after 2, 4, 6, and 24 h of exposure. Overall, Lso-carrying psyllids were more likely to settle on healthy tomatoes (*P* = 0.014), whereas the Lso-free psyllids settled on PVY-infected plants more frequently (*P* = 0.007). Asterisk indicates significant difference within observation time.

Settling of the Lso-carrying tomato/potato psyllids was overall affected by the presence of PVY infection (χ_1_^2^ = 6.02, *P* = 0.014), with the psyllids more frequently alighting on the healthy plants (Fig. 3). The choice to settle on healthy plants was consistent over time as revealed by the nonsignificant effect of time (χ_3_^2^ = 1.05, *P* = 0.789) and the nonsignificant interaction between the PVY status and time (χ_3_^2^ = 2.1, *P* = 0.552).

Interestingly, the Lso-free tomato/potato psyllids showed a significant overall preference toward PVY-infected plants (χ_1_^2^ = 7.28, *P* = 0.007). Although no effect of observation time was present (χ_3_^2^ = 3.72, *P* = 0.298), there was a significant time × PVY status interaction (χ_3_^2^ = 8.64, *P* = 0.034) indicating that the observed overall pattern was not consistent over time (Fig. 3). Lso-free tomato/potato psyllid preference for PVY-infected plants appeared to gradually increase over time and was statistically significant (sign test, *P* = 0.007) 24 h after release (Fig. 3).

### Variations in amino acids and sugars levels

**Figure 4:**
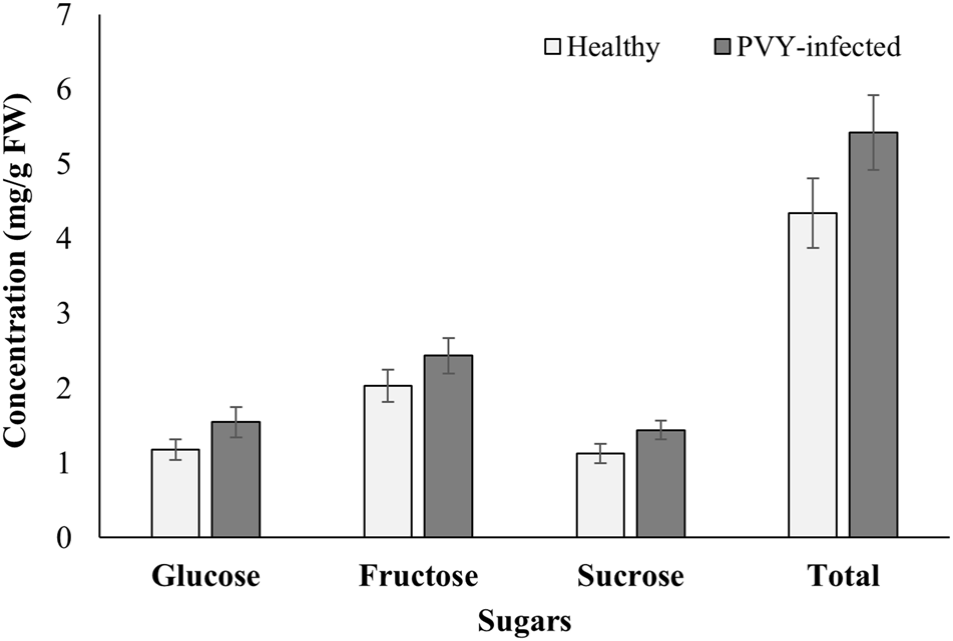
Sugar concentration in healthy and PVY-infected tomato plants. There were no significant effects of PVY infections on the sugar concentrations in tomato seedlings. Error bars represent standard error (± SE).

Total amino acid concentration was not significantly different between PVY-infected and healthy tomato hosts (F_1,16_ = 3.96, *P*_*adj*._ = 0.51); however, all of the evaluated amino acids consistently showed relatively higher concentrations in the PVY-infected plants. Except for serine (F_1,16_ = 32.35, *P*_*adj*._ = 0.003), none of the evaluated amino acid concentrations were significantly affected by the presence of PVY (Table 1). Concentrations of glucose (F_*1,14*_ = 2.29, *P*_*adj.*_ = 0.234), fructose (F_*1,14*_ = 1.54, *P*_*adj.*_ = 0.234) and sucrose F_*1,14*_ = 2.96, *P*_*adj.*_ = 0.234) were not influenced by the PVY infection (Fig. 4).

**Table 1:** Mean (± SE) concentrations (nmol/mg FW) of individual amino acids detected on tomato plants.

Amino acids	PVY°-infected	Healthy	*P*-value (adjusted)
Alanine	0.393 ± 0.040	0.288 ± 0.007	0.299
Glycine	0.133 ± 0.025	0.092 ± 0.017	0.561
Valine	0.183 ± 0.027	0.135 ± 0.016	0.561
Leucine	0.117 ± 0.016	0.084 ± 0.007	0.410
Isoleucine	0.108 ± 0.011	0.081 ± 0.006	0.432
Threonine	0.347 ± 0.035	0.243 ± 0.029	0.363
Serine	1.717 ± 0.199	0.720 ± 0.060	< 0.003*
Proline	2.092 ± 0.359	1.897 ± 0.422	0.561
Asparagine	0.572 ± 0.123	0.329 ± 0.086	0.553
Aspartic acid	1.147 ± 0.124	0.887 ± 0.111	0.561
Methionine	0.047 ± 0.003	0.038 ± 0.003	0.538
Hydroxyproline	0.201 ± 0.019	0.179 ± 0.012	0.561
Glutamic acid	3.006 ± 0.296	2.645 ± 0.241	0.561
Phenylalanine	0.136 ± 0.011	0.106 ± 0.007	0.410
α-aminoadipic acid	0.062 ± 0.010	0.028 ± 0.009	0.341
Glutamine	1.095 ± 0.180	1.025 ± 0.381	0.561
Glycine-proline (dipeptide)	0.075 ± 0.005	0.060 ± 0.006	0.488
Lysine	0.077 ± 0.013	0.060 ± 0.006	0.561
Histidine	0.072 ± 0.009	0.055 ± 0.008	0.555
Tyrosine	0.064 ± 0.008	0.043 ± 0.006	0.432
Tryptophan	0.069 ± 0.012	0.059 ± 0.015	0.561
Essential amino acids	1.157 ± 0.115	0.861 ± 0.079	0.352
Total amino acids	11.713 ± 0.990	9.055 ± 0.863	0.512

*Significant differences in levels of individual amino acids due to PVY°.

### Variations in phytohormone levels

**Figure 5:**
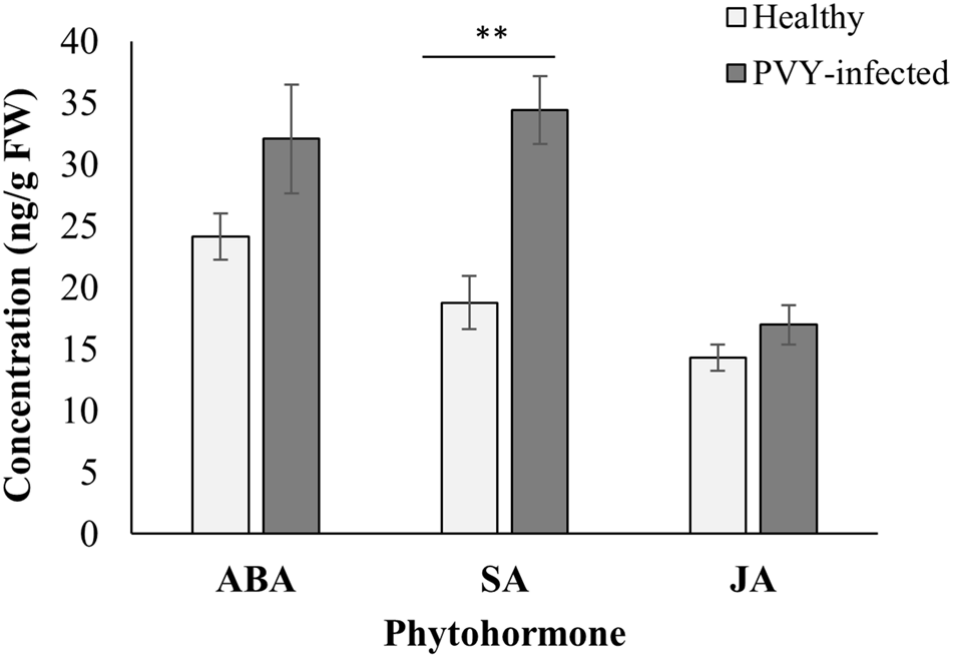
Phytohormone concentrations in healthy and PVY-infected tomato plants, with only SA showing a significant increase following PVY° infection; the significant difference is indicated by an asterisk (*P* = 0.003). Error bars represent standard error (± SE).

ANOVA revealed significant differences in SA concentrations, with PVY-infected host plants having significantly higher quantities than the healthy host plants (F_*1,14*_ = 20.08, *P*_*adj.*_ = 0.003). The levels of ABA (F_*1,14*_ = 2.72, *P*_*adj.*_ = 0.187) and JA (F_*1,14*_ = 1.92, *P*_*adj.*_ = 0.187) showed no significant responses to PVY infection (Fig. 5).

## Discussion

Studies of indirect, host-mediated interactions between pathogens and insects have become of greater interest, recently^4,53–56^. In the present study, the pre-existing PVY infection did not affect the inoculation or multiplication of Lso but triggered behavioral and biological responses from both the Lso-carrying and Lso-free tomato/potato psyllids (Fig. 1). The Lso-carrying psyllids exhibited increased oviposition and preference to settle on healthy leaflets (Figs. 2a and 3). In contrast, while no statistical difference was detected in oviposition by the Lso-free psyllids on PVY-infected and healthy tomato plants (Fig. 2b), they showed significantly higher preference to alight on the leaflets of the PVY-infected hosts (Fig. 3). Egg hatch rate was not influenced by the PVY status of the host plant in either Lso-carrying or -negative psyllids. The lack of statistical difference in egg hatch rates was not unexpected since abiotic environmental conditions such as temperature and relative humidity^57^ are among the most important factors influencing the hatch rate of tomato/potato psyllids.

Our post hoc quantification of plant biochemistry suggested that some of the observed differences in tomato/potato psyllid response to the PVY° infection may have been associated with changes in plant physiology. Infection with PVY° resulted in an increase in SA concentration (Fig. 5), a finding consistent with the results by Baebler et al.^58^ and Kersch-Becker and Thaler^59^ with the PVY^NTN^ strain of the virus. However, Kersch-Becker and Thaler^59^ did not report a significant increase in SA concentration after PVY° inoculation. The absence of a significant increase in SA level following PVY° infection in Kersch-Becker and Thaler^59^ may be due to the time of post inoculation sampling, i.e. ≤ 48 h. In our study, however, all physiological quantifications were conducted three weeks after PVY° inoculation. SA has been known to be associated with increased activity of several enzymes and compounds that are involved in plant defensive responses against pests^60^; examples include peroxidase, polyphenol oxidase, phenols, H_2_O_2_, various defense proteins^61^ and phenyl-propanoid compounds^60^. Reduced settling and oviposition of Lso-carrying psyllids on PVY-infected tomato plants could be explained by SA-dependent defensive responses following PVY infection. Interestingly however, neither the inoculation success of Lso by tomato/potato psyllids nor Lso titers were influenced by the elevated SA levels in the presence of PVY. One possible explanation would be that Lso may be capable of suppressing plant defenses, as reported in the closely related liberibacter species “*Ca.* L. asiaticus”, where the pathogen has been shown to utilize salicylate hydroxylase (SahA) to degrade SA^62^. Alternatively, or in addition, the success of SA-related defenses in limiting Lso multiplication may be short-lived since Lso can reduce the accumulation of transcripts related to SA synthesis^8^ over time.

Plant viruses are also known to alter the amino acid content in phloem or soluble carbohydrate content in leaves^20,34,37,38,40,58,59^, and those changes can be associated with herbivore attraction^42^ or repulsion^20^. In the present study, no notable effect of PVY infection was detected on glucose, fructose, or sucrose concentrations (Fig. 4). Despite a consistent relative increase across essential and nonessential amino acids observed in the PVY-infected tomato plants, serine was the only amino acid that had significantly higher concentrations in the PVY-infected host (Table 1). Our findings, however, differed from Prager et al.^20^ in the TMV-tomato pathosystem, where higher concentrations of amino acids and sugars were reported in noninfected than infected tomato hosts. This inconsistency indicates that some of the plant responses to infection may be virus specific. It is also important to note that not all amino acids have been shown to promote insect oviposition or development^63,64^. Additional studies are needed to draw more definitive conclusions on the impact of amino acids on tomato/potato psyllid oviposition and preference.

Interestingly, oviposition of the Lso-free tomato/potato psyllids was not influenced by PVY infection or the associated changes in SA and amino acids. Lso has been shown to negatively impact tomato/potato psyllid fecundity^65^. Therefore, it is possible that the Lso-carrying vectors avoided plants infected with an existing pathogen, as changes in the host physiology could further jeopardize their reproductive fitness. This, however, does not explain the observed preference of Lso-free tomato/potato psyllids to visit the PVY-infected hosts. There are several examples of host-mediated interactions where insect vectors, including the potato psyllid, at least temporarily showed preference toward infected plants^2,39^. The host cues responsible for this preference in the tomato/potato psyllid are not known, but it is possible that Lso-infected and PVY-infected plants had similar changes in olfactory and/or visual cues influencing settling behavior of psyllids. To our knowledge there has been no study on the potential role of tomato/potato psyllids as PVY vectors, and additional studies are warranted to explore underlying mechanisms of host-mediated interactions between PVY and non-aphid sap sucking insects.

Insect orientation and host preference are known to be driven by a combination of visual and olfactory cues from the host plant^66^. In the ZC pathosystem, the previously reported preference of Lso-carrying and Lso-free tomato/potato psyllids to settle on healthy or Lso-infected tomato plants, respectively, has been suggested to be driven by differences in plant volatile blends before and after Lso infection^67^. Similarly, in our study, the Lso-carrying tomato/potato psyllids showed a significant preference to settle on healthy tomato hosts, whereas the Lso-free psyllids settled more frequently on the PVY-infected hosts. Moreover, the similarities in the directional responses by the Lso-carrying and Lso-free psyllids toward Lso-infected tomatoes in Davis et al.^68^ and PVY-infected tomatoes in the present study suggested that both pathogens might modify host volatiles in ways that encourage greater vector visitation. We did not quantify changes in volatile profiles of the tomato seedlings following PVY inoculation, a topic which could be the subject of future studies.

Our results suggested that PVY infection can potentially impact the ZC pathosystem dynamics by affecting host preference and oviposition of potato/tomato psyllids. We refrained from statistically comparing Lso-carrying and Lso-free psyllid responses because bioassays were not performed simultaneously. However, it was important to discuss the observed patterns since Lso is known to impact both the tomato/potato psyllid fecundity^65^ and host preference^65,70^. Indeed, PVY infection in host plants led to different oviposition and host preference responses in Lso-carrying and Lso-free psyllids. The observed adverse effect of PVY infection on the oviposition of Lso-infected tomato/potato psyllid suggests that the presence of virus could potentially slow psyllid population growth within a field. However, we also observed that Lso-carrying psyllids prefer to alight on and deposit their eggs on healthy plants. Thus, in a PVY-affected field, the arrival of Lso-carrying psyllids is likely to promote the spread of liberibacter disproportionately among healthy plants, further reducing productivity of the agroecosystem. Finally, coinfections with different pathogens may contribute to the evolution of a pathogen's virulence, influencing plant disease dynamics^69^. Although we observed no effect of PVY infection on Lso inoculation success or multiplication, we have yet to examine the impact of PVY on Lso acquisition by the psyllids.

In summary, PVY infection had no significant impact on Lso inoculation and Lso titer. However, the presence of the viral infection in the host plant significantly reduced oviposition by, and host attractiveness, or possibly palatability, to the Lso-carrying vectors, whereas viral infection increased host attractiveness to the Lso-free tomato/potato psyllids. To our knowledge the potential role of tomato/potato psyllids in PVY spread has yet to be determined, and future studies are needed to evaluate implications of our findings in both PVY and Lso epidemiology. Further, there are multiple known strains of PVY and haplotypes of Lso and psyllid vectors, while we only focused on one vector and Lso haplotype and one relatively less virulent strain of PVY. Indeed, it has been demonstrated that the intensity of plant physiological responses to PVY infection is influenced by the virus strain^59^. Future studies are needed to investigate whether outcomes of the ecological interactions between ZC and PVY pathosystems are influenced by differences among Lso/vector haplotypes and/or PVY strains.

## Materials and methods

### Plant and insect material

All the experiments were conducted with the tomato variety Yellow Pear (*Solanum lycopersicum* L.; W. Atlee Burpee & Co. Warminster, PA). Tomato was selected as our biological model because, compared to potatoes, it is easier to maintain and manage in the greenhouse and laboratory. Tomato plants were grown from seed in seedling trays and later transplanted into 8.5 × 8.5 × 10-cm plastic pots filled with a soil mix of 70% sand, 20% peat moss (Sun Gro Horticulture Canada Ltd., Seba Beach, AB, Canada), and 10% vermiculite (Therm-o-Rock West INC., Chandler, AZ, USA) and slow-release fertilizer (Osmocote, N-P-K mix of 14-14-14; Scott-Sierra Horticultural Products Co., Marysville, OH, USA).

The PVY° strain (isolate PVY-Oz^70^; GenBank Accession No. EF026074) used for virus inoculations was from an infected tobacco (*Nicotiana tabacum* L.) source plant maintained in the greenhouse at the University of Idaho Aberdeen Research and Extension Center, Aberdeen, ID. The tobacco plants were maintained in a dome-shaped Bugdorm insect rearing cage (75 × 75 × 115-cm dimensions) (BugDorm Products, Taiwan) in the greenhouse. Temperatures alternated between 16 °C at night and 23 °C during the day with a photoperiod of 12 h. The virus strain was confirmed in tomato plants by polymerase chain reaction (PCR) analysis; viral cDNA was used as template for PCR analysis and the strain of PVY was identified using strain-specific primers^71–73^.

Haplotype B of Lso^74^ and the Central haplotype of tomato/potato psyllid^75^ were used in all experiments. The haplotype of the Lso-infected colonies was confirmed by PCR following the restriction analysis described in Swisher et al.^76^. The tomato/potato psyllid colonies were originally collected from infested potato and tomato plots in Weslaco, TX, USA, and were maintained in growth chambers with temperatures ranging between 18 and 27 °C, and a 16 h photoperiod, in the University of Idaho Integrated Pest Management (IPM) laboratory in Aberdeen Research and Extension Center, Aberdeen, ID. Each colony was reared inside a 60 × 60 × 60-cm tent-shaped Bugdorm cage (BioQuip Products, Rancho Dominguez, CA). Lso-carrying and Lso-free psyllids were reared on potato (*Solanum tuberosum* L., variety ‘Russet Burbank’). The infection status of psyllids was verified by PCR, according to the protocol by Crosslin and colleagues^77^.

### PVY inoculation

PVY inoculations were performed on tomato seedlings, approximately 12 cm in height (3–4 leaf stage). The inoculum was prepared by grinding the source plant (tobacco) leaf tissue in 0.1 M phosphate buffer (2:1), pH 7.5. Carborundum 400 grit (Beta Diamond Products, INC., CA, US) was used as the abrasive product. Then, 1 ml of the sap was gently rubbed on the surface of each of five leaflets of each plant, using a cotton ball^78^. Mock-inoculated plants were treated only with virus-free buffer solution and the abrasive product (Carborundum). After inoculations, tomato plants were maintained in the greenhouse inside 60 × 60 × 90-cm zip-front mesh cages (BioQuip, Rancho Dominguez, CA). Plants were maintained in the greenhouse with temperatures ranging between 16 °C at night and 23 °C during the day, on a 12 h photoperiod. Three weeks after PVY° inoculation, plants were used for experiments. PVY- and mock-inoculated plants were maintained in separate cages throughout the experiment to eliminate the possibility of accidental virus transmission through contact. PVY status was confirmed by PVY ImmunoStrip (Agdia, Elkhart, IN) at the end of each experiment, i.e., three, four, and six weeks after PVY inoculation for choice, no-choice, and transmission experiments, respectively. To sample, a fully developed young leaf, typically the 3rd leaf from the top of the plant, was collected (Supplementary material, Table S1). Only PVY-inoculated plants which tested positive for PVY (hereafter, PVY-infected) were included in data analyses. Mock-inoculated healthy controls (hereafter, healthy) never tested positive for PVY.

### "*Ca.* Liberibacter solanacearum" transmission and titer assays

Transmission assays were conducted on tomato plants three weeks after mechanical inoculations with PVY°. Two frameless leaf cages (BioQuip Products, Rancho Dominguez, CA), with inside dimension of 2.54-cm, were installed on two fully developed leaflets of each plant. To inoculate plants with Lso, two psyllids from the Lso-carrying colony were randomly selected and placed inside each leaf cage. The psyllids were allowed to feed for a 48-h inoculation access period. After the inoculation access period, psyllids and eggs were gently removed without damaging the leaflet and plants were maintained for three weeks in the greenhouse under aforementioned conditions. The collected psyllids and plant tissues (see below) were stored in − 20 °C prior to Lso quantification. Lso transmission success and Lso titer were compared between PVY-infected (*n* = 39) and healthy tomato plants (*n* = 29).

To determine Lso infection status and titer in the experimental plants, 100 mg of petiole tissue was collected from a fully developed young leaf from the upper third of the tomato plants three weeks after Lso inoculation. All of the tomato/potato psyllids and collected plant tissues were examined by quantitative PCR (qPCR; see below). The experiment was conducted in three time-blocks, with a minimum of 8 plant replicates per treatment in each time-block.

### Psyllid oviposition and egg emergence experiments

No-choice experimental assays were set up to evaluate the impact of PVY infection on the oviposition and hatch rate of Lso-carrying tomato/potato psyllid eggs. A minimum of 10 replicates per treatment was included in each of the two time-blocks. PVY-infected (*n* = 43) and healthy (*n* = 30) plants were established as previously described. Each plant was exposed to a total of four Lso-infected adult psyllids; two female/male pairs were caged on two different leaves for 48 h. The exact age of the adult psyllids was not known. Following the 48-h period, psyllids were collected, clip cages were removed, and eggs were counted. Plants were maintained in temperatures ranging between 20 and 27 °C, on a 16 h photoperiod and were inspected one week after exposure to the psyllids to count the number of the hatched nymphs. Hatch rate was calculated by dividing the number of hatched nymphs by the total number of eggs per plant. The number of eggs and viability (i.e., hatch rate) were compared between PVY-infected and healthy plants. A later no-choice experiment with PVY-infected (*n* = 24) and healthy plants (*n* = 19) was also conducted with Lso-free tomato/potato psyllids, using the approach outlined above.

### Psyllid host preference experiments

Experiments were conducted under controlled conditions with temperatures ranging between 20 and 27 °C, with a 16 h photoperiod. All assays were conducted in observation chambers made of two layers of foam of the following dimensions 19.5 × 12 × 1-cm (length × width × height) each with a 13 × 5.5 × 1-cm (length × width × height) opening in the middle (Supplementary file, Fig. S1). Before each observation, the petiole of the terminal leaflet of a PVY-infected and a healthy plant were placed between the two layers of foam at each of the two ends of the chamber, and were covered with a clear transparency sheet on one and transparent mesh on the other side, allowing only leaflets to be exposed inside the chamber. Two rectangle cardboard frames (19.5 × 12 cm) were used to cover and secure the mesh and transparency sheets on each side of the chamber. During evaluations the bottom of the arena was covered with a black 19 × 8-cm cardboard sheet to facilitate observations. See supplementary information for visual description of the observation chambers (Supplementary file, Fig. S1).

In each replicate, one adult Lso-carrying or Lso-free female tomato/potato psyllid was gently placed into the middle of the chamber through a hole in the middle of the mesh-covered surface of the observation chamber. Prior to being released into the observation chamber, each Lso-carrying/Lso-free female was placed with a Lso-carrying/Lso-free male inside a clear pure gelatin capsule (Capsuline size 1, FL. USA) for 24 h. The psyllid preference was recorded 2-, 4-, 6-, and 24-h post release. Preference was determine based on the psyllid presence on either PVY-infected or healthy leaflet at each recording time. The preference of Lso-free (*n* = 12) and Lso-carrying psyllids (*n* = 17) was evaluated separately.

### Lso titer quantification

Total DNA from both petiole and psyllid samples was extracted using the CTAB (hexadecyltrimethyllammonium bromide) method, following Buchman et al.^79^ and Marzachi et al.^80^, respectively. The DNA extraction quality and concentration were verified with a spectrophotometer (NanoDrop Lite, Thermo Scientific, Madison, WI). Lso was quantified by qPCR SYBRgreen using a CFX Real-Time PCR System (Bio Rad Laboratories, Hercules, CA, USA). The qPCR reaction contained 150 nM or 100 nM of each of the primers, HLBr and LsoF^81,82^ for psyllids and petiole tissue, respectively, 1X SsoAdvanced Universal SYBR Green Supermix (Bio Rad Laboratories, Hercules, CA, USA), and 150 ng of DNA template. The amplification program was set as follows: one cycle at 98 °C for 2 min, 40 cycles of 95 °C for 10 s, and 62 °C for 20 s, followed by a melt curve (65 °C to 95 °C, increment 0.5°Cs^−1^). Every qPCR plate included a negative control (DNA from healthy plants) and water control (no template control). Lso copy number was calculated according to Levy et al.^83^. A manufactured (SGI–DNA) plasmid (pIDTSMART–KAN) containing a 250 bp region amplified by primers HLBr and Lso was used for building the standard curve. The plasmid was diluted tenfold and dilutions were used to generate the standard curve. The number of copies was calculated for each standard dilution following the methods specified by Levy et al.^83^.

### Amino acids and sugars analysis

For sampling and extraction of leaves, eight individual plants were selected for each of the PVY-infected and healthy treatments. They all were tested for PVY status at the time of sampling using PVY ImmunoStrip (Agdia, Elkhart, IN). Two samples, 200 mg each, were removed from one fully developed young leaf from the top third of the plant. Each sample was transferred to a 1.5 mL cryo-tube, weighed and frozen in liquid nitrogen. All collected samples were stored at − 80 °C until extraction. To extract, samples were first milled using an Omni Bead Ruptor 12 homogenizer (Omni International Inc., Kennesaw, GA) for 60 s at high speed. Then, 1 mL of the extraction solvent, methanol:chloroform:water (8:1:1), was added to each tube^84^ and samples were ground for another 30 s at low speed. Extraction was performed for 1-h in a sonication ice bath (Bransonic Ultrasonic Corporation, Danbury, CT). The tubes were centrifuged at 21,000 g for 10 min at 4 °C, after which supernatant was collected and transferred into a 1.5 mL microcentrifuge tube. The extracts were dried under nitrogen flow and re-dissolved in 1 mL phosphate buffer saline. The average amino acid and sugar concentrations (see below) across the two sub-samples from each plant were used for comparisons between PVY°-infected and healthy tomatoes.

Twenty amino acids and one dipeptide including alanine, glycine, valine, leucine, iso-leucine, threonine, serine, proline, asparagine, aspartic acid, methionine, hydroxyproline, glutamic acid, phenylalanine, α-aminoadipic acid, glutamine, glycyl-proline (dipeptide), lysine, histidine, tyrosine, and tryptophan were detected and quantified in the samples. Amino acids were quantified by gas chromatography using a Shimadzu GC-FID system (GC-2014), equipped with an AOC-20 autosampler (Shimadzu, Kyoto, Japan). An EZ-FAAST kit, with a 10 m × 0.25 mm ZB-PAAC column, was used for extractions following Phenomenex protocol (Phenomenex, Torrance, CA, USA). The carrier gas (He) flow rate was kept constant during the run at 1.5 mL/min. For each sample (and standards) 2 μL was injected into the inlet heated to 300 °C, with a 1:15 split ratio. The oven was programmed at a ramp rate of 32 °C/min from 110 °C to 320 °C. The detector temperature was adjusted at 320 °C. External calibration curves were plotted for all the 33 amino acids included in the standards and used for the quantification.

Sugar composition and concentrations were analyzed by a high-performance anion exchange chromatography with a pulsed amperometric detection (HPAEC-PAD) system (Dionex ICS-5000, Sunnyvale, CA), a Dionex CarboPac PA1 column (2 × 250 mm) and a 2 × 50-mm guard column. An isocratic elution with 150 mM NaOH was used to separate glucose, fructose, and sucrose. The eluent flow rate was 0.25 mL/min. After each ten injections, the column was regenerated first by 150 mM NaOH and 500 mM sodium acetate, and then by 150 mM NaOH to prevent any shift in the peaks. External calibration curves were plotted for the three sugars for quantifications. Above mentioned extracts were diluted 5 times with ultrapure water and 10 μL of the samples and standards were injected to the system using an auto-sampler (Dionex AS-AP Autosampler, Sunnyvale, CA).

### Phytohormone analysis

The same eight plants that were used for amino acids and sugars analysis were separately sampled for phytohormone analysis. Two samples, 200 mg each, were removed from one fully developed young leaf from the top third of the plant. The leaf samples were transferred to cryo-tube, weighed, frozen in liquid nitrogen and stored at − 80 °C until extraction. To extract, the samples were milled using an Omni Bead Ruptor 12 homogenizer (Omni International Inc., Kennesaw, GA) for 60 s at high speed, then 1 mL of the extraction solvent (methanol:water; 7:3) with 150 ng internal standard was added to each tube for extraction according to Trapp et al. (2014)^85^. The samples were milled again for 30 s at low speed, and extraction was done for 30 min using a vial shaker at 800 rpm. The tubes were centrifuged at 21,000 g for 10 min at 4 °C, and the supernatant was removed and transferred into a 1.5 mL microtube. The pellets were washed with 200 μL of methanol and the supernatant was added to the microtubes. The extracts were dried in a SpeedVac Concentrator (Savant Instruments Inc. Farmingdale, NY), and re-dissolved in 100 μL methanol for the analysis. Average of the two half-leaves of each plant was used for statistical analysis as independent replicates.

The phytohormone analysis was performed using high-performance liquid chromatography (HPLC) on an Agilent 1200 Series HPLC system with a diode array detection (DAD) system coupled with an Agilent G1969A TOF–MS system equipped with an ESI source (Agilent, Santa Clara, CA, USA). A Zorbax XDB-C18, 50 mm × 4.6 mm, 1.8 µm column (Agilent, Santa Clara, CA, USA) was used for the separation holding at 30° C^86^. Each injection volume was 10 µL. The mobile phase consisted of 0.1% formic acid in water (solvent A) and 0.1% formic acid in methanol (solvent B), and the program started with a linear gradient from 50 to 95% B in 5 min, followed by isocratic elution at 95% B for 2 min, and then equilibrated at 50% B for 3 min according to Davis et al.^87^. Quantification was performed in the reconstructed ion current mode using m/z of 263.13 (abscisic acid), 137.02 (salicylic acid), and 209.11 (jasmonic acid), and corresponding external standards.

### Statistical analysis

Data were analyzed with IBM SPSS Statistics ver. 25.0. To evaluate the effect of PVY° on Lso inoculation success, a generalized linear mixed model (GLMM) with a binomial distribution and a logit link function was applied. The model included PVY status (PVY-infected or healthy plants) as the fixed factor and, block as the random factor. Lso status (0 = negative, 1 = positive) was used as the response variable.

Lso titers were log-transformed after a fixed value of 1.5 was added to all Lso concentrations to improve normality. A GLMM with a Gaussian distribution was used to compare Lso titer between PVY-infected and negative tomato plants. The Lso-inoculated plants that tested negative for Lso (i.e., “0” titer) were excluded from the analysis. The model included PVY status as the fixed factor, and block as the random factor.

The number of eggs on PVY-infected and healthy plants was compared with the non-parametric Mann–Whitney U test. Egg hatch rate was compared between PVY-infected and healthy plants, using GLMM with a normal distribution with PVY status as the fixed factor and block as the random factor.

The data from choice experiment was analyzed using a binary logistic regression. The observation time was treated as the repeated categorical measure. The model included PVY status, time of observation, time × PVY status interaction, and block as factors. In the case of Lso-free psyllids, there was a significant time × PVY status interaction and sign tests (GraphPad.com) were used to compare tomato/potato psyllid preference for PVY-infected and healthy leaves within each observation time. Direct comparisons in host choice and oviposition preference between Lso-carrying and Lso-free psyllids were not conducted since experiments were performed at different times.

Individual analysis of variances (ANOVA) were used to compare concentrations of amino acids, sugars, and phytohormone between PVY-infected and healthy plants. All values were log-transformed to meet normality and sequential Bonferroni correction was applied to all p-values (*P*_*adj*_) to adjust for multiple comparisons.

## Supplementary Information

Supplementary Information.

## Data Availability

The data used to support the findings of this study are available from the corresponding author upon request.
