# Author Correction: Interspecific interactions within a vector‑borne complex are influenced by a co‑occurring pathosystem

**DOI:** 10.1038/s41598-021-90072-2

**Published:** 2021-05-17

**Authors:** Regina K. Cruzado-Gutiérrez, Rohollah Sadeghi, Sean M. Prager, Clare L. Casteel, Jessica Parker, Erik J. Wenninger, William J. Price, Nilsa A. Bosque-Pérez, Alexander V. Karasev, Arash Rashed

**Affiliations:** 1 Department of Entomology, Plant Pathology and Nematology, University of Idaho, Aberdeen R&E Center, Aberdeen, ID 83210 USA; 2 Department of Entomology, Plant Pathology and Nematology, University of Idaho, Moscow, ID 83844 USA; 3 Department of Plant Science, University of Saskatchewan, Saskatoon, SK S7N 5A8 Canada; 4 Department of Plant Pathology and Plant-Microbe Biology, Cornell University, Ithaca, NY 14853 USA; 5 Department of Entomology, Plant Pathology and Nematology, Kimberly Research & Extension Center, University of Idaho, Kimberly, ID 83341 USA; 6 College of Agricultural and Life Sciences, Statistical Programs, University of Idaho, Moscow, ID 83844 USA

Correction to: *Scientific Reports* 10.1038/s41598-021-81710-w, published online 26 January 2021

This Article contains an error in Figure 3, where the color labels are reversed. The correct Figure 3 appears below as Figure 1.

**Figure 1:**
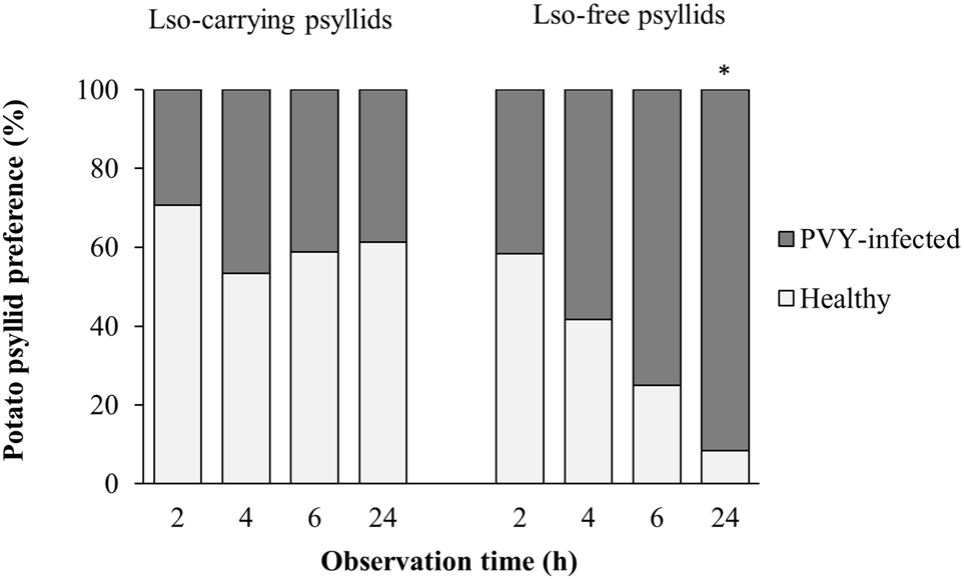
Percentage of Lso-carrying and Lso-free psyllids observed on healthy and PVY-infected tomato plants after 2, 4, 6, and 24 h of exposure. Overall, Lso-carrying psyllids were more likely to settle on healthy tomatoes (*P* = 0.014), whereas the Lso-free psyllids settled on PVY-infected plants more frequently (*P* = 0.007). Asterisk indicates significant difference within observation time.

