# Supplementary material for: Interspecific interactions within a vector-borne complex are influenced by a co-occurring pathosystem
Source: Sci Rep. 2021 Jan 26;11:2242. doi: 10.1038/s41598-021-81710-w (PMC7838419; doi:10.1038/s41598-021-81710-w)
Supplement: Supplementary file 1 — Supplementary Information. [file 41598_2021_81710_MOESM1_ESM.docx]

**Interspecific interactions within a vector-borne complex are influenced by a co-occurring pathosystem**

Regina K. Cruzado-Gutiérrez^1,2§^, Rohollah Sadeghi^2§^, Sean M. Prager^3^, Clare L. Casteel^4^, Jessica Parker^2^, Erik J. Wenninger^5^, William J. Price^6^, Nilsa A. Bosque-Pérez^2^, Alexander V. Karasev^2^ and Arash Rashed^1,2*^

^§^ Authors contributed equally

^1^ University of Idaho, Department of Entomology, Plant Pathology and Nematology, Aberdeen R&E Center, Aberdeen, ID 83210, USA

^2^ University of Idaho, Department of Entomology, Plant Pathology and Nematology, Moscow, ID 83844, USA

^3^ Department of Plant Science, University of Saskatchewan, Saskatoon, SK S7N 5A8, Canada.

^4^ Department of Plant Pathology and Plant-Microbe Biology, Cornell University, Ithaca, NY 14853, USA

^5^ University of Idaho, Department of Entomology, Plant Pathology and Nematology, Kimberly Research & Extension Center, Kimberly, ID 83341, USA

^6^ University of Idaho, College of Agricultural and Life Sciences, Statistical Programs, Moscow, ID 83844, USA

***** Corresponding: arashed@uidaho.edu

Figure S1. The setup of the observation arena used for the tomato/potato psyllid host choice bioassays (a) Schematic representation of the observation arena, (b) Assembled arena and experiment setup, (c) Recording of the potato psyllid preference.

**
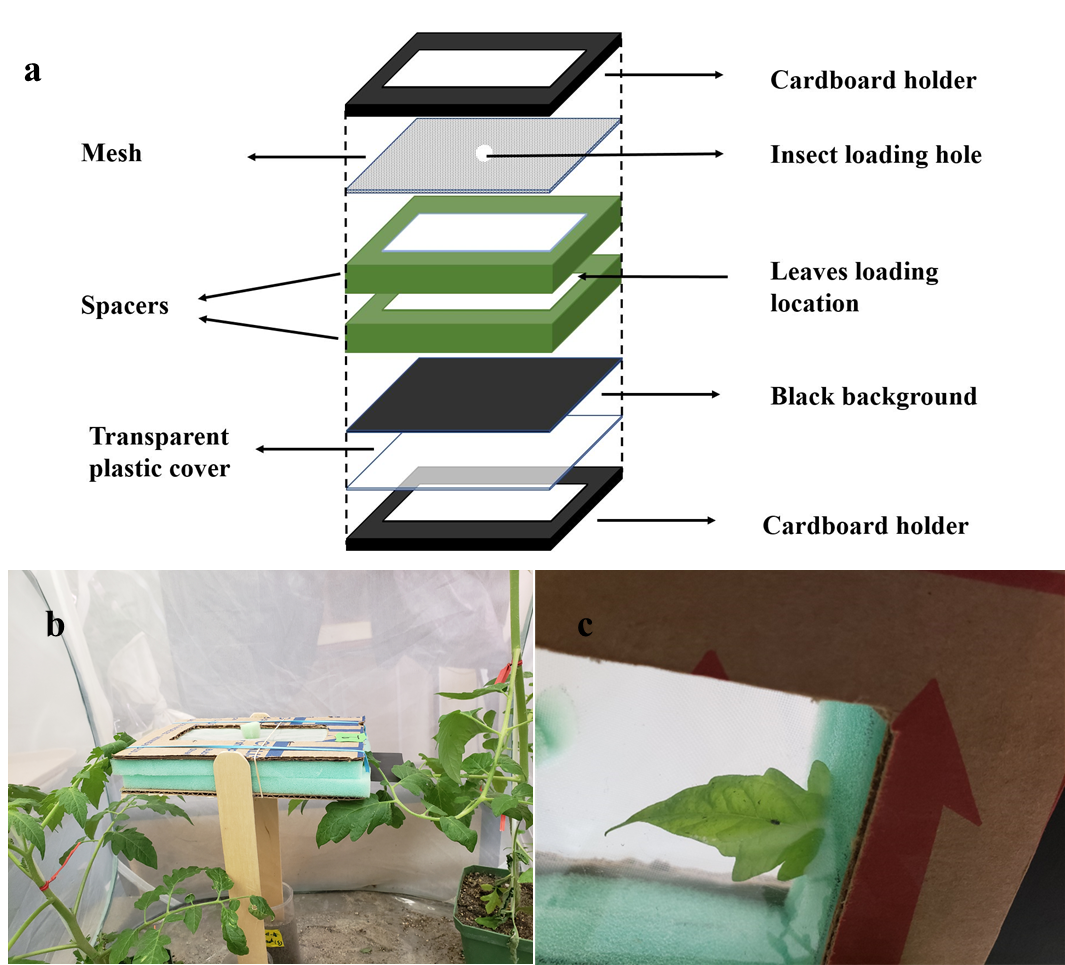
**

Table S1. Timeline of the events in choice, no-choice and transmission experiments.

| Procedure | stage | Week 0 | Week 1 | Week 2 | Week 3 | Week 4 | Week 5 | Week 6 |
| --- | --- | --- | --- | --- | --- | --- | --- | --- |
| PVY inoculation |  |  |  |  |  |  |  |  |
| Transmission assay: | Lso inoculation |  |  |  |  |  |  |  |
|  | Lso quantification |  |  |  |  |  |  |  |
|  | Confirming PVY status |  |  |  |  |  |  |  |
| No choice experiment: | Psyllid exposure |  |  |  |  |  |  |  |
|  | Egg counting |  |  |  |  |  |  |  |
|  | Hatch rate evaluation |  |  |  |  |  |  |  |
|  | Confirming PVY status |  |  |  |  |  |  |  |
| Choice experiment: | Evaluating responses/confirming PVY status |  |  |  |  |  |  |  |
